# Comparative study of eminectomy and use of bone miniplate in the articular eminence for the treatment of recurrent temporomandibular joint dislocation

**DOI:** 10.1016/S1808-8694(15)31282-9

**Published:** 2015-10-20

**Authors:** Álvaro B. Cardoso, Belmiro C.E. Vasconcelos, David M. de Oliveira

**Affiliations:** 1 Course of Specialization Bucco-Maxillo-Facial Surgery and Traumatology, Dental Sciences School, Universidade de Pernambuco (FOP/UPE); 2 Joint Professor and Coordinator, Master and Ph.D. Dissertation in Dental Sciences (Major in CTBMF), FOP/UPE; 3 Specialist in CTBMF, Master studies in Dental Sciences under course (Major in CTBMF), FOP/UPE

**Keywords:** temporomandibular joint, dislocations, mandible

## Abstract

Dislocation of the temporomandibular joint occurs when the mandibular condyle exits the glenoidal cavity and remains anteriorly locked to the articular eminence. It is repetitive (recurrent dislocation), usually associated with mandibular hypermobility and inclination of the articular eminence.

**Aim:**

This study intended to clinically and radiologically assess the technique of eminectomy and the use of a miniplate on the articular eminence for the treatment of recurrent dislocation of the temporomandibular joint of patients operated on at Oswaldo Cruz University Hospital from January to September 2003.

**Study design:**

Retrospective cohort.

**Material and Method:**

The sample consisted of 11 patients. Eminectomy was performed on nine joints of five patients and the placement of a miniplate on the articular eminence was performed on 11 joints of six patients. Data collection was carried out through analysis of patient's medical charts and new postoperative visit.

**Results:**

The results showed that there were no major postoperative complications with either technique. Maximum mouth opening was greater with eminectomy procedure and none of the patients operated on presented any recurrence of dislocation.

**Conclusion:**

It is concluded that both techniques were effective in the treatment of recurrent dislocation of the temporomandibular joint.

## INTRODUCTION

Currently, whenever referring to temporomandibular joint (TMJ), the term subluxation is referred to self-reducible displacement of mandible condyle anterior to articular eminence, which does not happen in luxation, in which it is required to reduce manually the displaced condyle to the glenoid cavity. In such a situation, pain stimulates spasms or mastication muscle contraction, which causes elevation and locking of condyle anterior to the articular eminence. Luxations or subluxations are normally bilateral, but they may also be unilateral.

In TMJ displacement (condyle luxation), the condyle is off its regular position, which may be anterior, posterior, superior, medial or lateral to the glenoid cavity. Anterior displacement is more common, and the other types are normally associated with fractures (Myrhaug, 1951).

According to Hale (1972), TMJ luxation occurs when the condyle moves outside the glenoid fossa, locking anteriorly to the articular eminence. This locking action is maintained by spasms of mastication muscles, inevitable leading to luxation. This conditions is named habitual, recurrent or relapsing when episodes become more frequent, worsening progressively. In such cases, it is associated with mandible hypermobility and inclination of articular eminence. Etiological factors of TMJ luxation are multiple and treatment varies from more conservative methods to complex surgical interventions.

Helman et al. (1984) reported two surgical treatment modalities for TMJ recurrent luxation: one intends to restrict mouth opening (increase in articular eminence by using a pad), and the other intends to promote free mandible movements (removal of articular eminence), each one with its own advantages and disadvantages.

Eminectomy, which consists of removal of articular eminence by ostectomy with use of rotation instruments associated or not with scalpels was first described by Myrhaug (1951). Since them it has been performed with satisfactory results and confirmed efficacy in the literature (Irby, 1957; Hale, 1972; Westwood, Fox, Tilson, 1975; Cherry, Frew Jr., 1977; Lovely, Copeland, 1981; Oatis, Baker, 1984; Helman et al., 1984; Progrel, 1987).

The use of a miniplate in the articular eminence is a more recent procedure whose main advantage compared to eminectomy is the fact that it is a reversible and less invasive method. Its main disadvantage is related to reduction of maximum mouth opening (Buckley, Terry, 1988; Puelacher, Waldhart, 1993). However, there are few publications in the literature that assess its use and there are no reports comparing it to eminectomy.

Thus, the present study aimed at assessing and comparing both techniques, given that they are the most frequently indicated for surgical approach of recurrent luxation of TMJ.

## MATERIAL AND METHOD

The study was conducted in the period between June 2003 and December 2003 in the city of Recife/PE. after the approval of the Research Ethics Committee, Universidade de Pernambuco (CEP/UPE). The studied population was gathered from medical files of Hospital Universitário Oswaldo Cruz (HUOC/UPE), which had been submitted to surgery for recurrent TMJ luxation with approaches of eminectomy and use of articular eminence miniplate, between the period of January 2001 and September 2003. It was a retrospective cohort study in which we assessed medical charts and protocols to learn about pre, trans and postoperative period. To reach higher reliability of data recorded in the medical charts, all patients were invited for a new visit.

The sample comprised 11 patients submitted to surgical treatment for recurrent luxation of TMJ. Six patients were operated on with titanium miniplate fixation on the articular eminence and five were operated using eminectomy technique, amounting to 22 surgeries (11 using miniplate and 9 using eminectomy). The number of procedures varied depending on history of luxation (uni or bilateral). We had a control group formed by patients submitted to eminectomy because it is the gold standard for this type of study, and the studied group, with patients submitted to surgery using miniplate.

The procedure followed the surgical protocol for treatment of recurrent luxation of TMJ using the Service of Bucco-Maxillo-Facial Surgery and Traumatology, HUOC/UPE. TMJ surgical approach for all patients was pre-auricular access.

**Figure 1:**
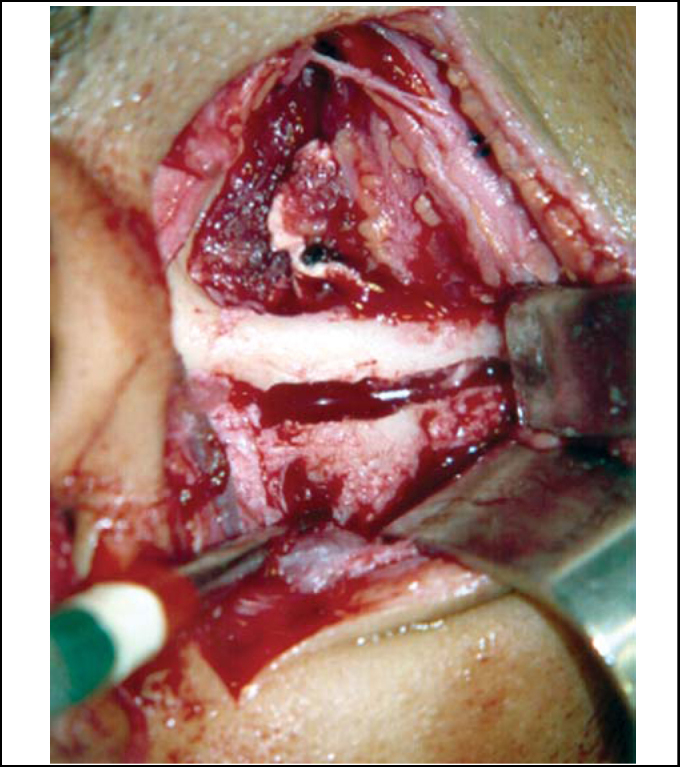
Demarcation of osteotomy in the articular eminence with 702 drill.

**Figure 2:**
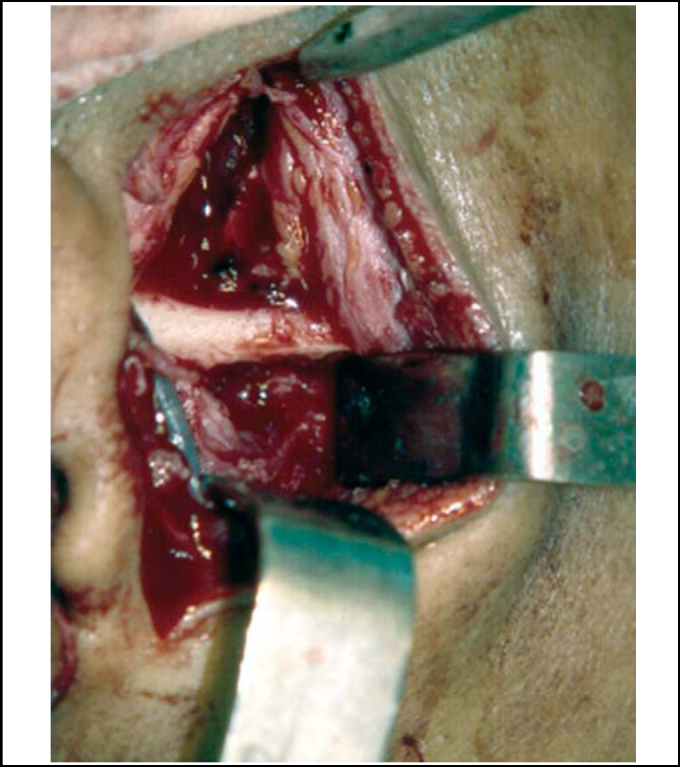
Bone regularization after removal of articular eminence.

To perform the technique of eminectomy, we marked the auricular eminence osteotomy using perforations with 702 drill, under continuous irrigation with distilled water, and upper limit of osteotomy was the lower margin of zygomatic arch (Figure 1). Osteotomy started with drill 702 along the length and depth of eminence, with inclination of approximately 10^o^ horizontal plan, and it was finalized with use of chisel and hammer. After removing the articular eminence, we performed bone regularization with pear-shaped multi-laminated drills (Figure 2). Functional mandibular movements were reproduced to confirm absence of luxation.

**Figure 3:**
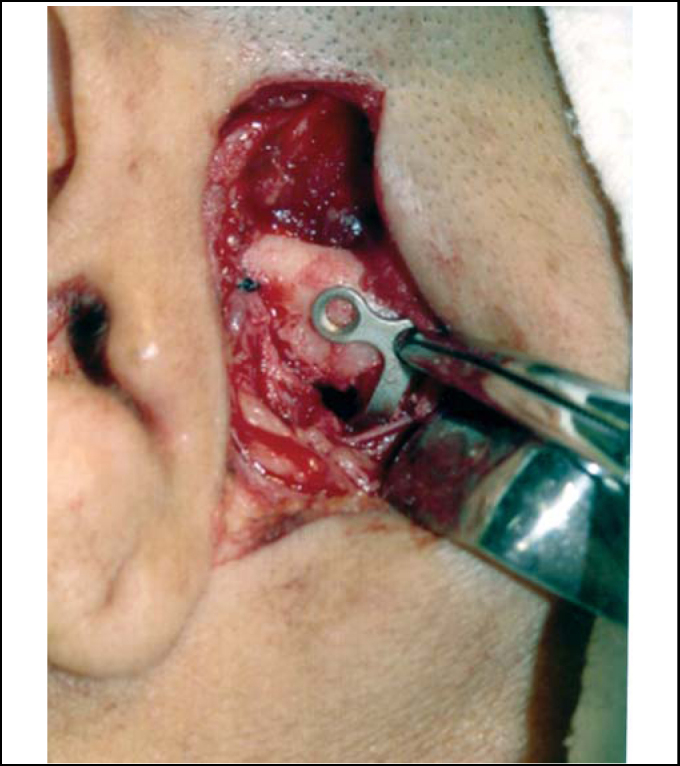
Positioning of L miniplate on the articular eminence considering condyle position.

**Figure 4:**
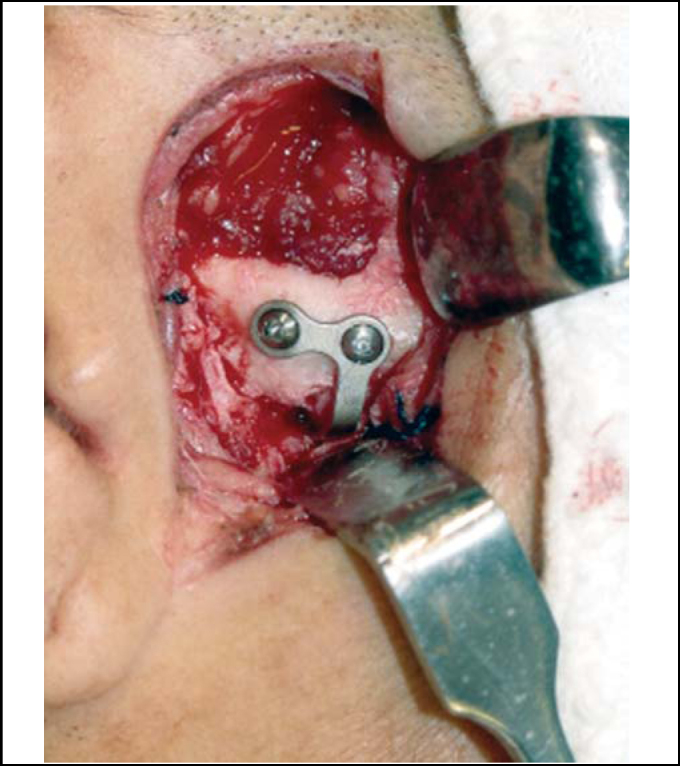
L miniplate fixed on the articular eminence after checking the best condyle position.

In order to place the miniplate, mouth opening was promoted up to the limit of condyle, reaching the lowest portion of articular eminence (reference for plate positioning), which led to approximately 40mm opening. Once the condyle was maintained in this position, we performed subperiosteal tunneling towards medial and inferior portions of articular eminence to find space to place the miniplate. We used titanium plates, system 2.0 L-shaped, with four holes and long intermediate portion (28.0mm longest segment of L), which was fixed with 6.0mm screws. The shortest segment of L was modeled to go laterally around the zygomatic arch and the longest segment was medially bent to be positioned inferiorly to articular eminence, taking into account the condyle position previously reached, aiming at promoting an increase in the eminence (Figure 3). The fixation of the smallest segment of L was made on the lateral surface of zygomatic arch with two 6.0mm screws; next, functional mandibular movements were made to confirm absence of luxation (Figure 4).

## RESULTS

Surgical treatment was made in 11 patients, five were male and six were female. Ages ranged from 18 to 40 years and the third decade of life was the most significantly affected. Patients presented recurrent TMJ luxation both unilateral (2 cases) and bilateral (9 cases). The treatment of choice was miniplate placement (6 cases) and eminectomy (5 cases). Patients were followed up for a period of 105 to 490 days, mean of 313.81 days of postoperative follow-up.

Surgical access used in all cases was pre-auricular, which amounted to 20 accesses - 9 bilateral cases and 2 unilateral cases. There were no cases of facial nerve damage in the studied sample; only 1 case of auricular-temporal nerve sensitive lesion that comprised bilateral anesthesia of the pre-auricular skin region, which was almost fully restored. In all cases, we recommended speech and hearing follow up, but only 6 patients out of 11 cases actually searched for specialized care.

**Graph 1:**
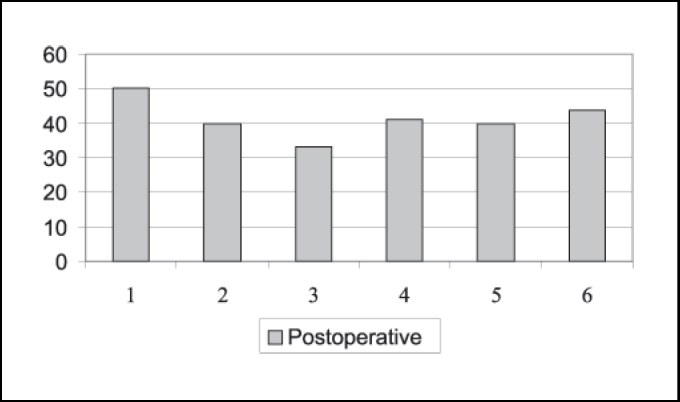
Number distribution of maximum mouth opening after surgery for each patient operated on with miniplate technique (mm).

**Graph 2:**
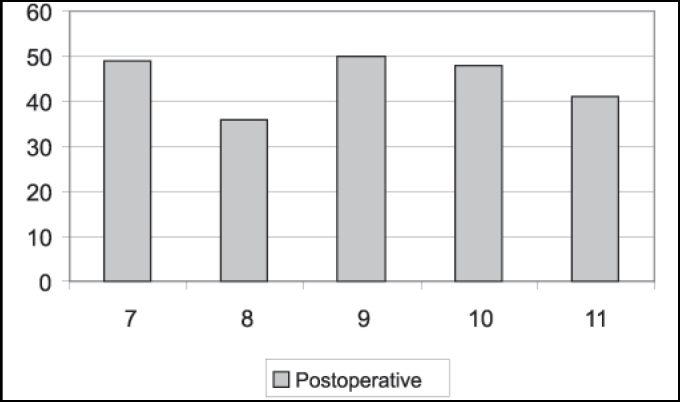
Number distribution of maximum mouth opening after surgery for each patient operated on with eminectomy technique (mm).

As to mandibular movements, we noticed that all patients presented laterality movements from the left to the right and protrusion both pre and postoperatively. In the preoperative assessment, all patients presented mandible hypermobility. Postoperatively, maximum mouth opening (ABM) ranged from 33.0mm to 50.0mm. Mean was 42.90mm. To patients operated using miniplate technique, ABM ranged from 33.0mm to 50.0mm, mean of 41.33mm, whereas ABM for patients operated on with eminectomy technique ranged from 36.0 to 50.0mm, mean of 44.8mm (Graphs 1 and 2).

**Graph 3:**
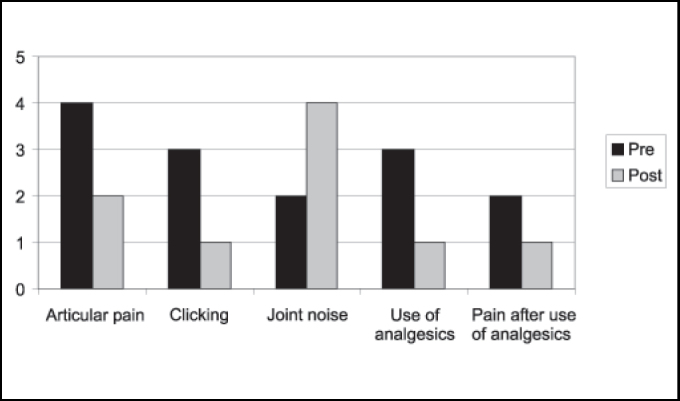
Distribution of patients submitted to miniplate technique concerning articular pain, clicking, and use of analgesic pre and postoperatively.

Considering the six patients operated on by the miniplate technique, four presented articular pain before surgery and three used analgesics. In two cases, pain improved with medication use. Postoperatively, there were two cases of articular pain. Only one of the patients used analgesics, with positive results. As to presence of clicking and noise in at least one of the sides of the joint pre and postoperatively, we detected reduction from three to one case concerning clicking and increase from two to four cases of noise (Graph 3).

**Graph 4:**
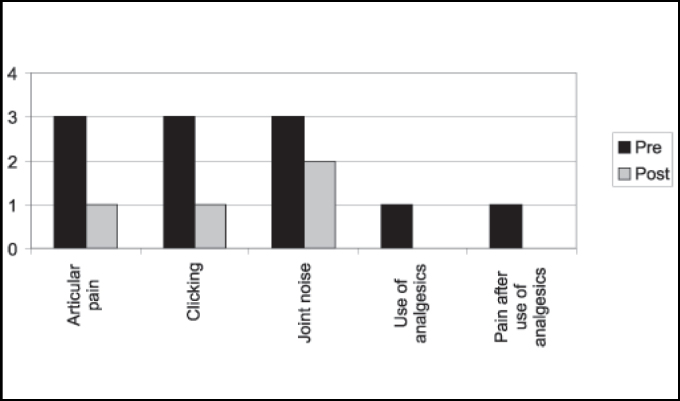
Distribution of patients submitted to eminectomy technique concerning articular pain, clicking, and use of analgesic pre and postoperatively.

Among the 5 operated cases using eminectomy, we could observe that three had preoperative articular pain and only one used analgesics to relieve symptoms. Postsurgically, we observed presence of articular pain in only one case and the patient no longer used analgesics. As to presence of clicking, in at least one of the sides, we detected reduction from three to one case postoperatively, which was similar for TMJ noise (Graph 4).

Patients were assessed concerning x-ray image of mandibular condyle preoperatively concerning presence of subchondral sclerosis, osteophytes and faceting. Out of 11 operated patients, there was one case of unilateral subchondral sclerosis, six patients presented faceting of condyle in at least one aspect, divided as two bilateral and four unilateral cases, and no cases of osteophytes. Postoperatively, we assessed the same categories previously referred and they were identical to that in the preoperative assessment. Imaging results of articular eminence did not evidence pre and postoperative bone affection.

As to recurrence, we did not observe postoperative recurrence in patients operated on with both techniques.

## DISCUSSION

Temporomandibular joint luxation represents 3% of all articular body luxations (Lovely, Copeland, 1981). However, many authors consider recurrent luxation as a rare condition. Similarly to other temporomandibular affections, the highest incidence of recurrent TMJ luxation is reported in female subjects, even though the reasons for this fact are still not fully understood (Myrhaug, 1951). It is also represented in our sample in which we detected higher incidence of women (55% when compared to men - 45%).

Anterior luxation of mandible is normally bilateral and symptoms include: inability to close the mouth, mentalis protrusion, tension and spasms of mastication muscles, excessive salivation, difficulty in phonation and pain in the TMJ region (Lovely, Copeland, 1981). In our study, we detected higher incidence of bilateral occurrence. However, we observed two patients that had unilateral luxation.

According to Shorey, Campbell (2000), many treatment modalities are considered in the resolution of pains and dysfunctions of recurrent TMJ luxation. In many cases, conservative methods promote some temporary relief of symptoms and recurrence is common. Surgical interventions are normally more effective for definite treatment. Reliable comparisons of the reports on modalities of treatment are difficult to find because of uneven periods of postoperative follow-up and different definitions of success rates. The authors found, among all different treatment modalities studied in the literature, a rate of 95% of cases without recurrence both after eminectomy and use of metallic implant over the articular eminence. In our study, we detected absence of postoperative luxation both with eminectomy and use of miniplate, even though the follow-up period had been variable.

Based on the assessment of mouth opening pre and postoperatively, Pogrel (1987) stated that the logics behind eminectomy seems to be suspicion, because the results shows reduction in maximum mouth opening after surgery, suggesting that the success of the treatment is more due to healing produced around the joint, which restricts anterior movement of condyle, because of the removal of articular eminence. However, in the 15 patients operated on by the author, he conducted unilateral eminectomy in all cases, both for unilateral and bilateral luxation, in which the operated site was the most affected one. In a minimum period of two years postoperative, there were no cases of recurrence. However, 11 cases presented deviation to the operated side during mouth opening movement. It is difficult to satisfactorily conclude about eminectomy based on cases operated unilaterally, considering that the side without surgical intervention may interfere in the results of the cases with bilateral luxation. Mouth opening before and after surgery may also be affected in this assessment. In our assessment, we compared directly maximum mouth opening (ABM) before and after surgery, and we detected satisfactory levels of ABM postoperatively (Graphs 1 and 2). We are aware, however, that patients may be afraid of luxation recurrence and in some cases it may significantly interfere in ABM.

Buckley, Terry (1988) have used since 1981 miniplate placement in the lateral side of the zygomatic arch with a segment bent medially right below the articular eminence. Compared to eminectomy, the authors consider the technique to be less invasive, reversible and does not require postoperative restrictions of mandible movements because of movement restrictions provided by the plate. It is believed to be the method of choice, especially in cases in which patients are not cooperative with the conservative treatment and for mentally disabled patients. The disadvantage is the reduction in maximum mouth opening with miniplate as opposed to eminectomy, but it is clinically insignificant. In the assessment of Graphs 1 and 2, we can observe a slight difference between postoperative ABMs between the miniplate placement technique and eminectomy, with lower indexes for the first one, but without clinical meaning. In Graphs 3 and 4, we assessed that both eminectomy and miniplate placement in the articular eminence were effective concerning articular pain, TMJ clicking and noise, except for the miniplate technique and noise, given that noise increased compared to preoperative results.

Speech therapy treatment did not present any correlation with the results using both techniques and concerning mouth opening, articular pain, TMJ clicking and noise, as well as the fact that imaging exams did not show bone affections at the condyle and articular eminence when comparing the two methods. According to the study, both techniques present positive results, without recurrence of TMJ luxation.

## CONCLUSION

1. Maximum mouth opening was higher in patients operated with eminectomy technique and other mandibular movements were present in all cases, regardless of the used technique.
2. Eminectomy technique proved to be more effective in relation to variable of TMJ noise and articular pain.
3. There was similar improvement in clicking variable for both techniques.
4. There were no imaging findings of articular components associated with the techniques.
5. Both techniques proved to be effective for the treatment of recurrent TMJ luxation and there were no further cases of recurrence.
